# Antibody-based targeting of alternatively spliced tissue factor: a new approach to impede the primary growth and spread of pancreatic ductal adenocarcinoma

**DOI:** 10.18632/oncotarget.7955

**Published:** 2016-03-07

**Authors:** Dusten Unruh, Betül Ünlü, Clayton S. Lewis, Xiaoyang Qi, Zhengtao Chu, Robert Sturm, Ryan Keil, Syed A. Ahmad, Timofey Sovershaev, Mariette Adam, Patrick Van Dreden, Barry J. Woodhams, Divya Ramchandani, Georg F. Weber, Janusz W. Rak, Alisa S. Wolberg, Nigel Mackman, Henri H. Versteeg, Vladimir Y. Bogdanov

**Affiliations:** ^1^ College of Medicine, University of Cincinnati, Cincinnati, OH, USA; ^2^ Leiden University Medical Center, Leiden, The Netherlands; ^3^ The Arctic University of Norway, Tromsø, Norway; ^4^ Diagnostica Stago R & D, Gennevilliers, France; ^5^ Haemacon Ltd, Bromley, UK; ^6^ College of Pharmacy, University of Cincinnati, Cincinnati, OH, USA; ^7^ McGill University Health Centre, Montreal Children's Hospital, Montreal, Canada; ^8^ University of North Carolina at Chapel Hill, Chapel Hill, NC, USA

**Keywords:** pancreatic cancer, tissue factor, alternative splicing, β1 integrins, metastasis

## Abstract

Alternatively spliced Tissue Factor (asTF) is a secreted form of Tissue Factor (TF), the trigger of blood coagulation whose expression levels are heightened in several forms of solid cancer, including pancreatic ductal adenocarcinoma (PDAC). asTF binds to β1 integrins on PDAC cells, whereby it promotes tumor growth, metastatic spread, and monocyte recruitment to the stroma. In this study, we determined if targeting asTF in PDAC would significantly impact tumor progression. We here report that a novel inhibitory anti-asTF monoclonal antibody curtails experimental PDAC progression. Moreover, we show that tumor-derived asTF is able to promote PDAC primary growth and spread during early as well as later stages of the disease. This raises the likelihood that asTF may comprise a viable target in early- and late-stage PDAC. In addition, we show that TF expressed by host cells plays a significant role in PDAC spread. Together, our data demonstrate that targeting asTF in PDAC is a novel strategy to stem PDAC progression and spread.

## INTRODUCTION

Pancreatic ductal adenocarcinoma (PDAC) survival rate remains dismal, with 5-year survival < 5%, and by 2030 is predicted to be the third leading cause of cancer-related death [1]. Pancreatic cancer is commonly associated with thrombotic events, which contribute to its morbidity and mortality [2, 3]. Cancer coagulopathy is thought to be partially due to the up-regulation of Tissue Factor (TF), the primary initiator of blood clotting [4, 5]. Multiple oncogenic events that are characteristic of PDAC, e.g. activation of the proto-oncogene K-RAS and inactivation/loss of p53 and PTEN, promote TF expression [6]. Recently, we reported that the secreted isoform of TF, termed alternatively spliced TF (asTF), is expressed in early-stage PDAC lesions (pancreatic intraepithelial neoplasia, PanIN) and abundant in advanced PDAC, in contrast to normal pancreas [7]. Our groups recently demonstrated that asTF acts on cancer cells in an auto/paracrine manner via β1 integrins, promoting disease progression by fueling cancer cell proliferation, survival, metastatic spread, neovascularization, and monocyte accumulation in the tumor stroma [7, 8].

Unlike the much-studied major form of TF termed full-length TF (flTF), asTF is minimally coagulant, and triggers intracellular signaling through non-proteolytic mechanisms by binding to α6β1 and αvβ3 integrins, and thereby activating PI3K/Akt, MAPK, and FAK pathways [9]. Integrin subunits α6 and β1 are up-regulated in PDAC, and play a crucial role in promoting PDAC progression [10]. asTF-β1 integrin interaction on microvascular endothelial cells increases the expression of cell adhesion molecules VCAM-1, ICAM-1, and E-selectin, which facilitates monocyte recruitment; we note that this effect is exerted by human and murine asTF [11, 12]. Levels of asTF positively correlate with the number of tumor associated monocytes/macrophages (TAMs), which are known to significantly contribute to tumor progression and resistance to chemotherapy [7–13]. Tumor cell-derived asTF fuels PDAC primary growth and spread via upregulation of various signaling pathways [7]; however, it is not known whether host-derived TF also plays a discernible role in PDAC pathobiology. In addition, it is not known whether tumor cell- and/or host-derived asTF can be targeted with an asTF-specific monoclonal antibody to stem PDAC progression.

In this study, we i) examined the asTF-integrin nexus as a potential therapeutic target in PDAC, including the activity of our novel, asTF-specific neutralizing antibody RabMab1; ii) delineated the mechanisms underlying asTF-induced PDAC progression; and iii) elucidated the relative significance of host-derived TF in PDAC. We employed a doxycycline (Dox)-inducible asTF transgene system to rule out the possibility of cell line and/or clonal selection variability influencing experimental outcomes. Using this approach, we investigated whether delayed-onset upregulation of asTF yields a phenotype that is distinct from that obtained via constitutive overexpression. The effectiveness of antibody-based targeting of asTF in PDAC was assessed *in vivo* using an orthotopic mouse model.

## RESULTS

### asTF-integrin interactions promote PDAC cell migration

**Figure 1:**
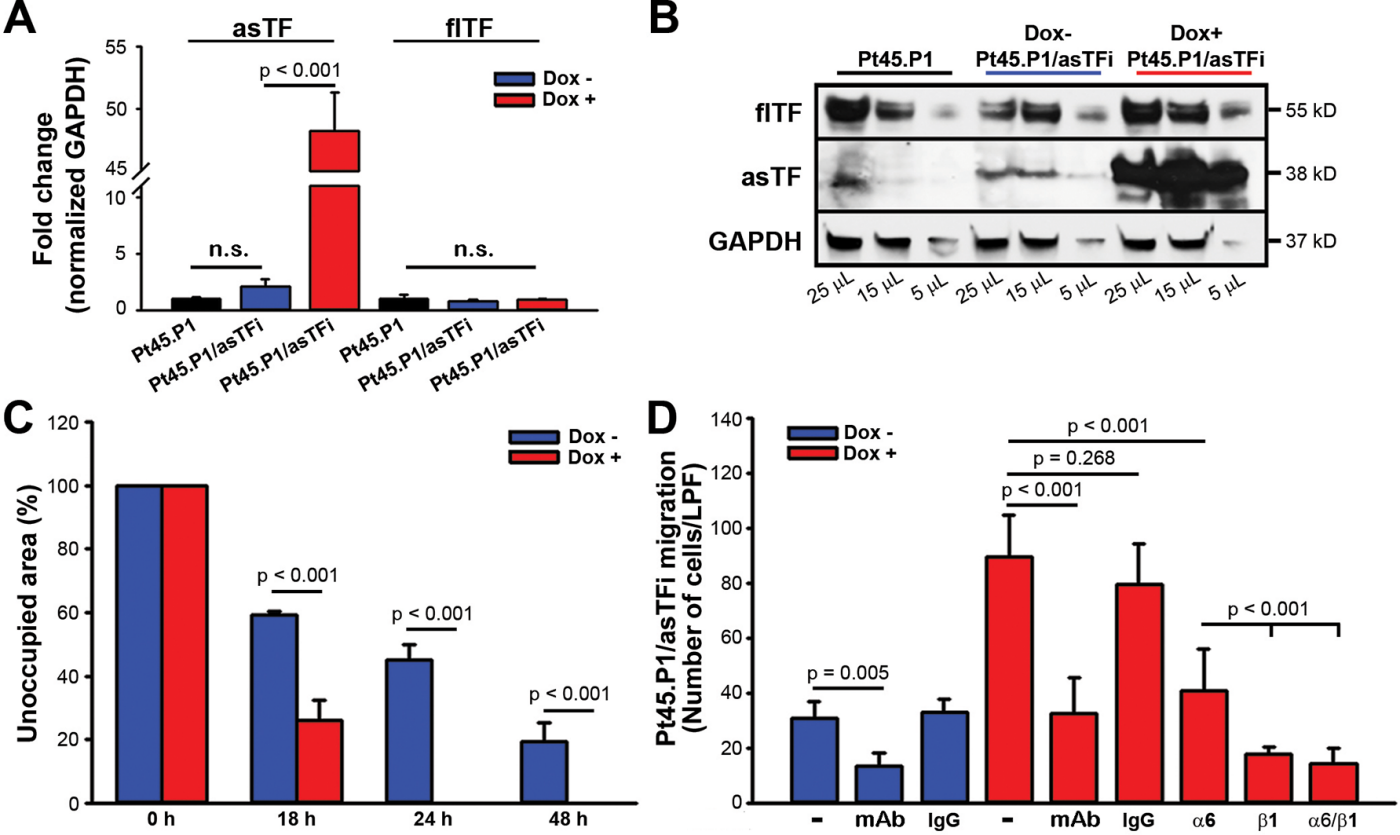
TF isoform expression in Pt45.P1/asTFi cells (**A**) asTF/flTF mRNA expression levels were assessed by quantitative real-time RT-PCR (*n* = 3). (**B**) Western blot, flTF/asTF protein levels in Pt45.P1 and Pt45.P1/asTFi cells; lysates were assessed for total protein concentration and volumes were adjusted accordingly. (**C**) Quantification of gap closure/scratch assay, Pt45.P1/asTFi cells treated and untreated with Dox. Bars depict the area unoccupied by Pt45.P1/asTFi cells (*n* = 3) at 0, 18, 24, and 48 hours. (**D**) Pt45.P1/asTFi cell migration toward serum in a transwell assay: laminin-coated transwell inserts were seeded with Pt45.P1/asTFi cells treated as indicated (*n* = 3 transwells per treatment; RabMab1 = mAb).

We recently reported that constitutive asTF overexpression in human pancreatic cancer cells (Pt45.P1) promotes metastatic spread *in vivo* [7]; here we sought to investigate the mechanisms responsible and specifically whether asTF increases cell motility. We engineered Pt45.P1 cells to inducibly express asTF (Pt45.P1/asTFi); when treated with Dox, Pt45.P1/asTFi cells had significantly higher levels of asTF mRNA and protein, while flTF mRNA and protein levels remained unchanged (*p* < 0.001) (Figure 1A, 1B). A scratch assay showed that Dox-treated Pt45.P1/asTFi cells had completed gap closure by 24 hours, whereas untreated cells still had unoccupied area at 48 hours (Figure 1C). Because asTF- α6/β1 integrin interactions promote breast cancer cell proliferation [8], we sought to determine whether this enhanced scratch closure was mainly due to enhancement of PDAC cell migration rather than cell proliferation; thus, we performed a 5-hour cell migration assay under a serum chemo-gradient using laminin-coated transmembrane inserts and Pt45.P1/asTFi cells. Laminin is abundantly expressed in PDAC stroma and is known to bind α6β1 integrins [10, 14]. As in the scratch assay, Dox-treated cells exhibited a significantly higher migration rate compared to untreated cells. Notably, when untreated Pt45.P1/asTFi cells were pre-incubated with the inhibitory anti-asTF antibody RabMab1, their basal migration rate was significantly reduced (Figure 1D), indicating that even the relatively low basal levels of asTF constitutively expressed in Pt45.P1/asTFi cells significantly contribute to their migratory potential. Pre-incubating Pt45.P1/asTFi Dox+ with anti-α6 inhibitory antibody yielded a partial reduction of cell migration, whereas pre-incubation with anti-β1 or anti-β1/anti-α6 fully inhibited cell migration (Figure 1D). Thus, asTF expressed in PDAC cells facilitates their integrin-mediated motility, a hallmark of PDAC progression and metastasis.

### asTF promotes primary growth and spread *in vivo* at early and later stages of tumor development

**Figure 2:**
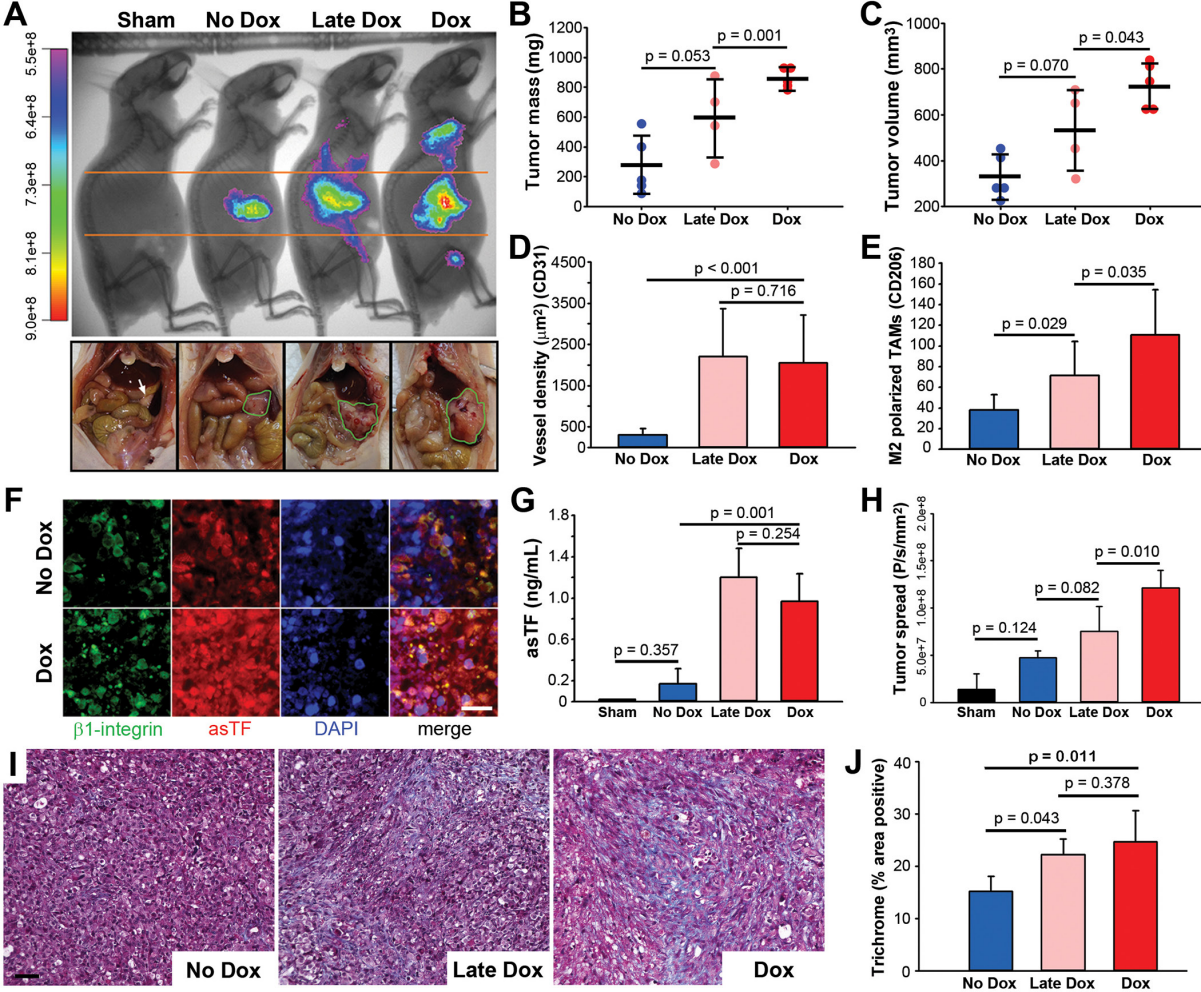
Growth of orthotopically implanted Pt45.P1/asTFi cells in nude mice (**A**) Mice began receiving Dox (2 μg/mL) in sucrose at day 1 of the study (“Dox”), day 25 of the study (“late Dox”), or sucrose alone (“No Dox”), and tumor progression imaged by CVM-SapC-DOPS 5 weeks post-surgery (*n* = 5/cohort; top row, representative images). Bottom row: abdominal cavities of nude mice bearing orthotopic Pt45.P1/asTFi tumors. White arrow: normal pancreas; green outlines: tumor. Quantification of tumor mass (**B**) and tumor volume (**C**) in the three groups (*n* ≥ 4). (**D**) 8 view fields per specimen (*n* = 5 per specimen type) were counted and averaged for vessel density as assessed by anti-CD31 staining. (**E**) Levels of M2 polarized TAMs in the stroma. (**F**) Expression and co-localization of β1 integrin and asTF in tumors formed by Pt45P1/asTFi cells (scale bar = 20 μm). (**G**) Plasma from mice bearing Pt45.P1/asTFi tumors was assayed for asTF using ELISA (*n* = 5). (**H**) Quantification of tumor spread to distal sites in anesthetized mice via CVM-SapC-DOPS imaging (analyzed areas were above and below the orange lines). (**I**) Representative images, Masson's Trichrome stain (scale bar = 50 μm). (**J**) Quantification of percent area positive for Masson's Trichrome stain, 5 view fields per specimen (*n* = 5).

To examine the temporal effect of asTF overexpression on tumor progression *in vivo*, we orthotopically implanted 1 × 10^6^ Pt45.P1/asTFi cells into the pancreata of nude mice (*n* = 5/group) and allowed tumors to develop for 5 weeks. Mice received Dox (2 μg/mL) in sucrose drinking water at day 1 (“Dox”), day 25 (“Late Dox”), or sucrose alone (“No Dox”), and tumor progression was monitored *in vivo* using CVM-SapC[H2]-DOPS imaging (Figure 2A). At 2.5 weeks post-implantation, no differences in tumor take and/or metastatic spread were observed between the cohorts (data not shown). At the end of the experiment, tumor growth was observed in all mice except one animal in the “Late-Dox” cohort. No appreciable distal metastases were observed in the “No Dox” cohort compared to the other two cohorts; distal spread was significantly reduced in “Late Dox” mice compared to “Dox” mice (*p* = 0.010), yet it was in-trend higher in “Late Dox” mice compared to “No Dox” mice (*p* = 0.082) (Figure 2A, 2H). Mice were then euthanized and primary tumors resected and examined for weight and volume. “Dox” tumors were significantly larger in both mass and volume compared to “Late Dox” and “No Dox” tumors (Figure 2B, 2C). These observations indicate that elevated expression of asTF can promote PDAC progression during early as well as late stages of the disease, yielding larger tumors and increased spread.

### Upregulation of asTF expression alters the composition of tumor stroma

Next, we compared the histology of “No Dox”, “Late Dox”, and “Dox” Pt45.P1/asTFi tumors for vessel density (CD31) and the levels of stromal M2-polarized tumor associated macrophages (TAMs) (CD206). While both “Late Dox” and “Dox” tumors had significantly increased vessel density compared to “No Dox” tumors, vessel density of “Late Dox” tumors was comparable to that of “Dox” tumors, which suggests that asTF-potentiated PDAC vascularization plateaus relatively early (Figure 2D). “No Dox” tumors also had significantly fewer TAMs, as did “Late Dox” tumors when compared to “Dox” tumors (Figure 2E). Consistent with our *in vitro* results obtained with Pt45.P1/asTFi cells, immunohistochemical analysis of tumor tissue revealed an appreciable increase of asTF expression in “Dox” vs “No Dox” tumors; extensive co-localization of asTF and β1-integrin was observed (Figure 2F). Remarkably, asTF protein was present in the circulation of mice in “Late Dox” as well as “Dox” cohorts at levels exceeding 1 ng/mL (Figure 2G). Lastly, we analyzed the tumors for the levels of collagen deposition (Masson's trichrome): “Late Dox” and “Dox” tumors had comparable collagen deposition, which was significantly higher compared to that in “No Dox” tumors (Figure 2I and 2J). Cumulatively, these findings suggest that asTF plays a multifaceted role in tumor stroma development, whereby it increases the levels of TAMs, vessel density, and collagen deposition.

### Targeting asTF impedes PDAC progression

**Figure 3:**
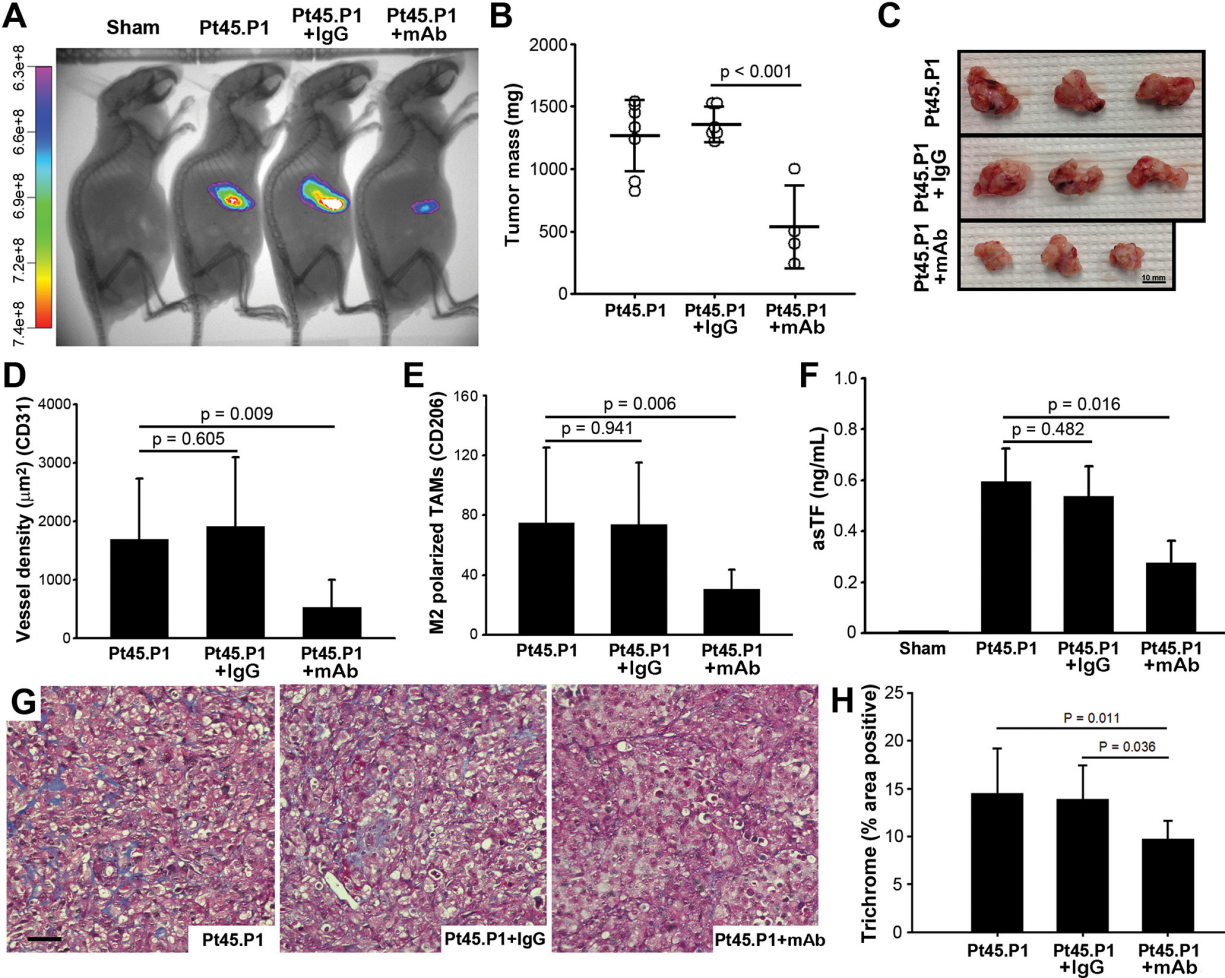
Effects of RabMab1 on the growth of orthotopically implanted Pt45.P1 cells in nude mice (**A**) Tumor progression was monitored *in vivo* via CVM-SapC-DOPS imaging over 7 weeks (*n* ≥ 3). (**B**) Quantification of primary tumor mass (*n* ≥ 3). (**C**) Representative specimens, resected tumors. (**D**) Eight view fields per specimen (*n* = 3 per specimen type) were assessed for vessel density, and (**E**) for M2 polarized TAMs. (**F**) Plasma samples from each cohort bearing Pt45.P1 tumors were assayed for asTF using ELISA (*n* = 3). (**G**) Representative images, Masson's Trichrome stain (scale bar = 50 μm). (**H**) Quantification of percent area positive for Masson's Trichrome stain. (RabMab1 = mAb).

To assess whether exposure to RabMab1 can stem the growth of PDAC cells expressing native levels of asTF in the orthotopic setting, as we found to be the case for breast cancer cells [8], we co-implanted Pt45.P1 cells with RabMab1 or isotype control IgG using the same cell number and RabMab1 quantity that we previously employed in our breast cancer studies, i.e. 100 μg/6 × 10^5^ cells, and tumor growth was monitored periodically using CVM-SapC[H2]-DOPS *in vivo* imaging over 7 weeks (*n* = 8/cohort) (Figure 3A). Tumors co-implanted with RabMab1 had a lower take compared to other cohorts (RabMab1: 4/8; IgG: 6/8; PBS: 7/8). Like PT45.P1/asTFi cells grown in mice not receiving Dox, Pt45.P1 cells did not exhibit significant spread even when grown for 7 weeks; when co-implanted with RabMab1, Pt45.P1 cells produced much smaller tumors (*p* < 0.001) (Figure 3B, 3C). Tumors produced by RabMab1-treated Pt45.P1 cells were also significantly less vascularized and had ~3.5 fold fewer M2 polarized TAMs compared to the tumors produced by Pt45.P1 cells co-implanted with vehicle (PBS) and/or isotype control IgG (Figure 3D, 3E). Mice in the Pt45.P1/RabMab1 cohort had a ~2 fold decrease in the levels of circulating asTF compared to mice in the Pt45.P1/PBS and/or isotype IgG cohorts (Figure 3F, 0.27 ng/mL vs 0.60/0.54 ng/mL, respectively; *p* < 0.001). Collagen deposition in the tumor stroma was also significantly reduced by RabMab1 (Figure 3G, 3H). Thus, antibody-based targeting of asTF is likely to comprise a viable therapeutic strategy to stem PDAC progression.

### Host TF contributes to PDAC progression

**Figure 4:**
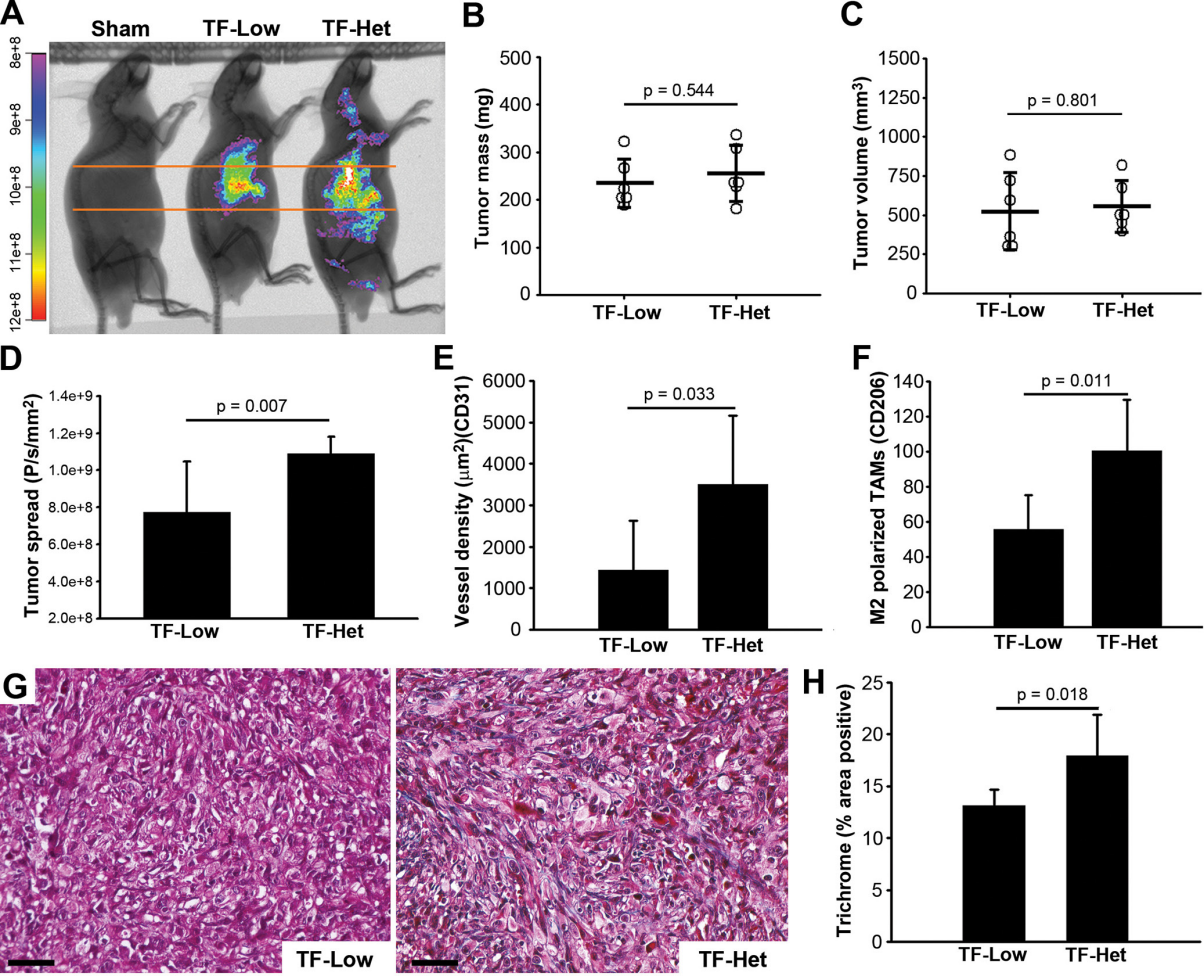
Contribution of host-derived TF to tumor progression, orthotopically implanted Pt45.P1 cells (**A**) Tumor progression was monitored via CVM-SapC-DOPS (*n* = 6). (**B**) Quantification of average primary tumor mass and (**C**) volume in each cohort (*n* = 6). (**D**) Quantification of spread to distal sites via CVM-SapC-DOPS imaging (analyzed areas were above and below the orange lines). (**E**) Eight view fields per specimen (*n* = 6 per specimen type) were assessed for vessel density, and (**F**) M2 polarized TAMs. (**G**) Representative images, Masson's Trichrome stain (scale bar = 50 μm). (**H**) Quantification of percent area positive for Masson's Trichrome stain.

We next sought to evaluate the contribution of host TF to PDAC growth and spread in our model using TF- Het (control) and TF-Low SCID mice. TF-Low mice express low levels of flTF but no asTF [15, 16]; thus, phenotypic changes observed in TF-Low mice can be due to either a reduction in host flTF and/or a reduction in host asTF. In light of our findings in nude mice pointing to a limited spread of Pt45.P1 cells in the setting of 1 × 10^6^ cells grown for 5 weeks (Figure 2) and/or 6 × 10^5^ cells grown for 7 weeks (Figure 3), we attempted to facilitate systemic spread in this experiment by orthotopically implanting 1 × 10^6^ Pt45.P1 cells into TF-Low/Het mice and allowing tumors to develop over 7 weeks. In agreement with prior studies pointing to SCID mice as a better platform to study the metastatic capacity of human tumor cells compared to nude mice [17], we indeed observed significant spread of Pt45.P1 cells in TF-Het mice (Figure 4A, 4D). The fraction of mice with detectable lung metastasis was higher in TF-Het mice than in TF-Low mice (4/6 vs 1/6). Pt45.P1 tumors grown in TF-Low/Het mice were comparable in mass and volume (Figure 4B, 4C); however, tumor spread was significantly diminished in TF-Low mice (*p* = 0.007) (Figure 4A, 4D). Tumors in TF-Low mice also had lower vessel density (*p* = 0.033) and had fewer M2 polarized TAMs in the stroma (*p* = 0.011) (Figure 4E, 4F). Fibrin(ogen) levels in the normal pancreata of TF-Het mice were comparable to those in TF-Low mice; in the tumors grown in TF-Het as well as TF-Low mice, fibrin(ogen) staining was prominent at the invasive edge/capsule and in necrotic cores (Supplementary Material), which is in agreement with prior observations reported for lung lesions [18]. Notably, TF-Low tumors had decreased collagen deposition compared to TF-Het tumors (*p* = 0.018) (Figure 4G, 4H). These results indicate that host-derived TF likely contributes to PDAC spread.

### RabMab1 suppresses tumor growth equally well in TF-Het and TF-Low mice

**Figure 5:**
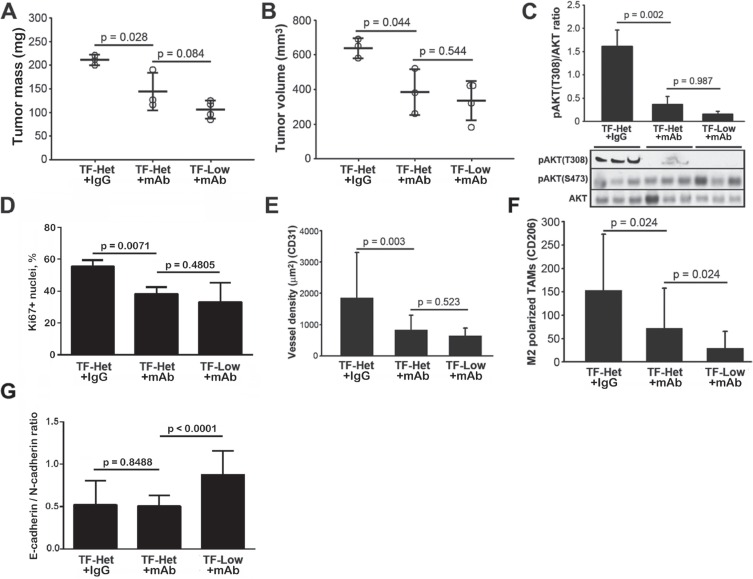
RabMab1 suppresses orthotopic growth of Pt45.P1 cells with equal efficacy, yet differentially affects the levels of tumor associated M2 polarized TAMs in TF-Het and TF-Low mice (**A**) Average primary tumor mass, TF-Low and TF-Het SCID mice bearing orthotopic Pt45.P1 tumors (*n* ≥ 3). (**B**) Average primary tumor volume (*n* ≥ 3). (**C**) Western blot, phosphorylation of AKT (T308 & S473) in the three cohorts; each lane is a sample of an individual tumor. (**D**) Quantification of percent of Ki67+ nuclei, (**E**) vessel density, (**F**) levels of M2 polarized TAMs, and (**G**) E-cadherin/N-cadherin ratio in the tumors grown in the three cohorts; a minimum of eight view fields per each tumor (*n* ≥ 3 per specimen type) were counted and averaged. (RabMab1 = mAb).

To compare the relative contribution of tumor-derived asTF and host flTF/asTF to tumor progression in our model, we analyzed the growth of Pt45.P1 cells co-implanted with RabMab1 or isotype control IgG in TF- Low and TF-Het mice. In the presence of RabMab1, Pt45.P1 cells implanted in TF-Low as well as TF-Het mice grew tumors that were significantly smaller compared to control tumors (Figure 5A, 5B). When grown in TF- Het mice, RabMab1 treated tumors had significantly more TUNEL positive nuclei (*p* = 0.0475); there was no difference in the percent of TUNEL positive nuclei in the tumors grown in TF-Het and TF-Low mice (data not shown). We previously reported that constitutive overexpression of asTF in PT45.P1 cells induces MAPK and Akt phosphorylation [7]; in this study, MAPK p42/44 phosphorylation levels were unaffected by RabMab1 treatment (data not shown), yet a highly significant decrease in pAKT-T308 was observed (*p* = 0.002), while the levels of pAKT-S473 were unaffected (Figure 5C). RabMab1 reduced the percent of Ki67+ positive nuclei and tumor vascularization equally well in TF-Het and TF-Low mice (Figure 5D, 5E), which likely indicates that, compared to host TF, tumor cell derived asTF is a more significant contributor to PDAC progression. Interestingly, RabMab1 suppressed the levels of M2 polarized TAMs in the tumors grown in TF-Low mice more potently than it did in TF-Het mice (Figure 5F); in agreement with that, E/N cadherin ratio was significantly increased in the tumors grown in TF-Low mice compared to the other two groups (Figure 5G). The levels of asTF protein expressed by tumor cells were not appreciably affected by RabMab1 (data not shown).

## DISCUSSION

In this study, we report the following set of findings: i) asTF-β1 integrin interactions render PDAC cells significantly more motile; ii) RabMab1-mediated targeting of asTF inhibits basal as well as asTF-potentiated migration of PDAC cells; iii) host flTF and/or asTF contributes to PDAC spread and influences the stromal composition of primary PDAC tumors; iv) tumor-derived asTF is cumulatively a more significant contributor to PDAC progression compared to host-TF.

Since the approval of gemcitabine in 1996, development of novel approaches to treat patients suffering from PDAC has remained stagnant. Over the last several years, different formulations and combinations of cytotoxic agents have improved the median survival rate by 4.4 months over gemcitabine monotherapy [19]. Despite these advances, there remains a severe need for new therapeutic modalities that can target host immunity, stromal microenvironment, and cancer cell signal transduction pathways [19]. In earlier studies, Rauch and colleagues reported that asTF is overexpressed in lung cancer and may thus contribute to its pathobiology [20, 21]; more recently, our groups demonstrated the potential for asTF to be a promising target in breast cancer [8]; in the present study, we show that inhibition of asTF-integrin interactions holds much promise in stemming PDAC progression. asTF promotes migration of PDAC cells; we posit that asTF-β1 interactions may render PDAC cells more motile via asTF competing with laminin for β1 integrin binding as well as promoting outside-in integrin signaling. It is also possible that, as we recently described in the breast cancer setting, asTF modulates binding and/or migration on laminin, similarly to how flTF modulates migration on laminin [8]; an even more intriguing scenario might be envisioned whereby asTF competitively removes flTF-dependent inhibition of β1 integrins on laminin. We note that asTF binds to a distinct region of β1 integrins (residues 579–799) thereby promoting a conformational change in β1 integrins that renders them prone to activation by other external ligands [8]. Our asTF-specific inhibitory monoclonal antibody RabMab1 inhibits basal and asTF-potentiated PDAC cell migration. Here we show that, in addition to promoting tumor cell migration, asTF alters the tumor microenvironment by increasing the levels of M2 polarized TAMs, vessel density, and collagen deposition in the stroma. In the presence of RabMab1, Pt45.P1 cells grew significantly smaller tumors with fewer TAMs, blood vessels, and reduced collagen content; future studies will address whether systemic and continuous administration of RabMab1, as opposed to cancer cell co-implantation, will have a major impact on PDAC progression: given the hypovascular, scar-like structure of desmoplastic PDAC tumors, the ability of antibodies to penetrate solid tumors remains a concern. Currently, we are defining the protein domain(s) of human asTF critical to its binding to β1 integrins: molecular mapping of asTF-β1 integrin interactions will likely aid in the development of short inhibitory peptides and/or small-molecule compounds with high tissue penetrance capacity.

There is considerable interest in therapeutic targets that can attenuate aberrant signaling transduction pathways in PDAC. The PI3K/Akt pathway mediates a variety of cellular processes, such as cell proliferation, survival, and motility [22]. The PI3K/Akt pathway is activated in PDAC, and targeting the PI3K/Akt pathway can overcome resistance to apoptosis-inducing chemotherapy [23]. Our previous studies showed that asTF-integrin interactions promote AKT phosphorylation [7, 9]. RabMab1 inhibits phosphorylation of Akt at T308, which suggests that a reduction in tumor growth and spread may be in part due to the suppression of the Akt-dependent pro-survival pathways. Interestingly, we did not observe a change in the levels of pAkt-S473 post-treatment with RabMab1; the relative abundance of pAkt-S473 and pAkt-T308 is known to differ between various PDAC cell lines [24] yet no definitive information is available as to the relative importance of either site in PDAC pathobiology. Still, a recent study examining the pAkt-S473/pAkt-T308 status demonstrated that, compared to pAkt-S473, enhanced phosphorylation of pAkt-T308 as a more reliable biomarker for the progression of head-and-neck small cell carcinoma induced by smoking and/or excessive alcohol consumption [25], the two well-established risk factors for PDAC [19].

Numerous *in vivo* studies and preclinical findings suggest that targeting macrophage recruitment, polarization and activation may also prove effective therapeutic strategies in PDAC and other solid malignancies [26]. In many forms of cancer, high densities of TAMs are associated with poor clinical outcomes [27]. Levels of M2-polarized TAMs expressing mannose receptor CD206 [28, 29], but not CD68, positively correlate with negative outcome. In addition to being a more selective TAM marker, CD206 is associated with immunosuppression and the release of a variety of tumor-promoting growth factors that correlate with poor prognosis [30, 31]. In our orthotopic PDAC mouse model, RabMab1 lowers the levels of stromal CD206^+^ TAMs and impedes systemic spread; we propose that amelioration of metastases may in part be due to a decrease in TAMs because M2 polarized TAMs are known to promote epithelial-mesenchymal transition of pancreatic cancer cells [28]. Of note, Pt45.P1 cells formed much larger tumors in nude mice compared to SCID mice and so it is reasonable to propose that genetic background likely affects tumor size as well as spread of pancreatic cancer cells in an orthotopic setting. Tumor cell TF-induced coagulation, which results in fibrin formation, may promote formation of pre-metastatic niches by rendering distal sites more receptive to tumor cell growth via the recruitment of CD11b^+^ monocytes/macrophages [32]. In that light, our findings shown in Figure 4 indicate that in PDAC, host TF (i.e. non-tumor cell associated TF) may contribute significantly to tumor spread, complementing the studies by Palumbo and colleagues who demonstrated that tumor cell associated TF contributes to metastatic seeding *in vivo* [33]. We note that our studies are also consistent with the findings of Yu and colleagues who showed that subcutaneous tumors formed in TF-Low mice have reduced vessel density which does not significantly affect primary tumor growth [15]; however, distal spread was not studied in their models. CD11b^+^ monocyte recruitment is mediated through the binding of CD11b to clot components and activated endothelium [34]; we note that asTF-integrin interactions on human as well as murine microvascular endothelial cells promote monocyte recruitment [11, 12].

PDAC is characterized by extensive desmoplasia and much of the primary tumor mass is comprised of (extra)cellular components other than cancer cells, some of which may serve as a source of host TF in the tumor microenvironment. The relative contribution of host vs tumor-derived TF to PDAC progression is unknown. In our study, severe depletion of host TF had no significant impact on primary tumor weight and/or volume; however, we did observe a significant decrease in vessel density and M2 polarized TAMs in the tumors grown in TF-Low mice. Most strikingly, systemic spread was also dampened in TF-Low mice. Still, targeting of tumor-derived asTF had a much more significant effect on PDAC progression compared to lowering the levels of host TF. Our data strongly suggest that asTF likely acts as an important regulator of the inflammatory microenvironment, known to play an essential role in K-RAS driven progression of PDAC [35]. In our hands, Pt45.P1 cells exhibit low constitutive levels of asTF but cooperate with exogenous asTF and host TF in formation of pro-inflammatory, pro-metastatic, collagen-rich, and desmoplastic milieu. It is possible that in addition to cancer cells themselves, inflammatory cells, and blood vessels [9], asTF also regulates the activity of pancreatic stellate cells implicated in PDAC-related fibrosis [36]. Because fibrosis and inflammation are regarded as elements of therapeutic intractability/resistance of PDAC to cytotoxic and targeted therapies [37], our results suggest that our anti-asTF monoclonal antibody RabMab1 could act as therapeutic sensitizer.

Aside from improving PDAC treatment, it is also critical to identify reliable circulating biomarkers that can non-invasively determine whether surgical intervention is feasible (e.g. resectable/unresectable disease), or aid in patient stratification. Multiple studies show that increased levels of TF protein circulating in plasma e.g. microvesicle-associated flTF, asTF, and/or degraded flTF [38] are associated with an increased risk for thrombosis in patients with cancer [39]. However, it is unclear whether circulating “total TF” comprises a cancer biomarker with prognostic utility. Using our novel asTF-specific ELISA, we show here that asTF is detectable at high levels in the circulation of mice bearing orthotopic PDAC tumors. Importantly, reduction of tumor size by RabMab1 led to a 3-fold reduction of the circulating levels of asTF from 600 pg/mL to ~200 pg/ml (Figure 3): last year, we reported that pre-operative circulating levels of asTF ≥ 200 pg/ml may help identify PDAC patients with a more aggressive disease [40]; thus, we plan to conduct a prospective study to determine whether measuring circulating asTF may also aid in detecting recurrence in PDAC.

In sum, our findings show that asTF-β1 integrin interactions play a major role in pathobiology of PDAC, and that antibody-based targeting of asTF may thus comprise a novel strategy to stem PDAC progression. Because asTF is i) dispensable to normal blood clotting, ii) expressed at higher levels in malignant tissues, and iii) able to promote tumor progression, it may very well comprise the preferred TF isoform to target in a cancer setting.

## MATERIALS AND METHODS

### Cell lines

Human PDAC cell line Pt45.P1 is classified as grade III, harbors K-RAS/p53/p16 mutations, and has been extensively characterized [41]/authenticated by short tandem repeat analysis (ATCC) [7]. A second, new cell line termed Pt45.P1/asTFi was generated by stably co-transfecting Pt45.P1 cells with i) linearized pTet-On Advanced vector, and ii) pTRE-Tight vector (both from Clontech) with a human asTF expression cassette cloned into the plasmid's multiple cloning site. Fugene HD (Roche) was used to transfect the constructs; cells were maintained in G418 and seven G418-resistant clones were harvested, expanded, and the consistency of the levels of doxycycline (Dox)-inducible asTF mRNA/protein overexpression was verified before the clones were pooled (data not shown). Both cell lines were grown in DMEM (Cellgro) supplemented with 10% fetal bovine serum (HyClone), 100 IU/mL penicillin, 100 μg/mL streptomycin, and 0.25 μg/mL amphotericin at 37°C in a humidified incubator (5% CO_2_). Neither primary growth nor spread of Pt45.P1 cells was affected by Dox (data not shown).

### *In vivo* studies

All animal studies were carried out in compliance with the protocol approved by the Institutional Animal Care and Utilization Committee, University of Cincinnati. Pt45.P1/asTFi cells were implanted in the pancreata of 5-week-old female nude athymic mice, (*n* = 5/cohort) (Harland Laboratories). Mice were subdivided into three cohorts: 1) animals that began receiving Dox (2 μg/mL in water/sucrose) on day 1 of the study (“Dox”), 2) day 25 of the study (“Late Dox”), and 3) sucrose water alone (“No Dox”) for 5 weeks. Pt45.P1 cells were implanted in the pancreata of nude mice with WT levels of murine TF, as well as mice rescued from embryonic lethality caused by deficiency of the murine TF gene (*Cf3*) with a human TF (hTF) mini-gene: rescued mice (mTF^−/−^, hTF^+/−^, hereafter TF-Low) express only ~1% of TF levels compared to 50% expressed by their heterozygous counterparts (mTF^+/−^/hTF^+/−^, hereafter TF-Het), and were backcrossed into severe combined immunodeficiency (SCID) background (*n* = 6/cohort) [15, 16].

*In vivo* efficacy studies of RabMab1 were carried out as follows. Pt45.P1 cells were resuspended in PBS containing 100 μg of RabMab1 or rabbit IgG isotype control, and implanted in the pancreata of nude athymic/TF-Low/Het mice. Tumor progression was monitored via SapC-DOPS imaging over 7 weeks or when otherwise specified as previously described (*n* = 8/cohort) [42]. In brief, a multispectral imaging system FX (Kodak) was used to visualize tumor growth and spread employing Cell-Vue Maroon (CVM)-labeled, nanovesicle-coupled Saponin C protein fragment H2 (CVM-SapC[H2]-DOPS) injected via tail vein; this compound selectively binds to tumor cells as well as tumor vasculature enriched in externalized phosphatidylserine [42]. Quantification of tumor spread was analyzed with Carestream MI software, background fluorescence normalized to sham-operated mice, and fluorescence was converted to photons per second per mm^2^. At the end of the study animals were sacrificed, necropsy was performed, and tumors were harvested and processed for protein, RNA, and histological analysis.

### Quantitative RT-PCR

Total RNA was isolated from cells and tissue flash-frozen in LN_2_ using RNeasy kit (Qiagen) as per the manufacturer's instructions; cDNA was synthesized using Transcriptor (Roche). Quantitative RT-PCR was performed using our validated TaqMan probe and primer sets for asTF and flTF [7]; GAPDH was used as a housekeeping gene.

### Western blotting

Cells were trypsinized, washed with PBS, and pelleted. Cell pellets were resuspended in PBS supplemented with Protease Inhibitor I cocktail (Roche) and lysed in Laemmli buffer with 2-Mercaptoethanol. Samples were denatured at 95°C for 10 min, loaded on 12% polyacrylamide gels (Life Technologies) and transferred to PVDF membranes. Membranes were blocked overnight at 4°C and probed with primary and corresponding HRP-conjugated secondary (Invitrogen) antibodies. Blots were developed using LumiLight (Roche), and chemiluminescent bands were visualized with X-ray film. Anti-pAkt T308 (Cat. No. 13038), anti-pAkt S473 (Cat. No. 9271), anti-total Akt (Cat. No. 9272), anti-total MAPK (Cat. No. 9102), and anti-pMAPK p42/44 (Cat. No. 9101) antibodies were from Cell Signaling.

### Cell migration studies

Gap closure/scratch assay was performed in triplicates by seeding 12-well plates with Pt45.P1/asTFi cells at 1 × 10^5^/well. Dox (final concentration: 2 μg/mL) was added to the medium to induce asTF a day before the scratch was made, and the expression levels of asTF were verified by Western blotting. After cells adhered and reached confluence, the wells of the plate were scratched at the center using a P200 pipet tip. The generated gaps were analyzed for closure at 0, 18, 24, and 48 hours. The results were quantified using Image J software (NIH) by measuring the area (in pixels) that remained unoccupied at each time point; the area at 0 hours was set to 100%.

Transmigration assays were carried out using 24- well plates and inserts with 8.0 μm diameter pores (BD Bioscience) pre-coated with 50 μg/mL laminin (Sigma-Aldrich Cat. No. L4544) in Hank's Balanced Salt Solution (HBSS; Cellgro) for 1.5 hrs. Excess laminin was removed from the inserts, following which 7.5 × 10^4^ Pt45.P1/asTFi cells were pre-incubated with anti-asTF antibody RabMab1, anti-integrin β1 (R & D Systems), anti-integrin α6 (R & D Systems), or isotype control antibodies (Jackson ImmunoResearch) for 30 min, placed in the upper chamber and allowed to migrate for 5 hrs at 37°C, 5% CO_2_ toward serum-containing medium in the lower chamber. Afterwards, the inserts were fixed in ice-cold methanol and non-migrated Pt45.P1/asTFi cells were removed from the luminal side with a cotton swab. Inserts were excised, stained, and preserved with Vectashield containing DAPI (Vector Labs). Images were captured on fluorescent microscope Keyence BZ-9000 (BIOREVO) at 20X; 6 representative fields per insert (*n* = 3 inserts per each experimental condition) were captured and results analyzed using Image J.

### ELISA

Platelet-poor murine plasma derived from arterial blood was assayed for asTF protein levels using our custom sandwich ELISA as described [43]; in brief, samples were placed in 96-well capture plates and incubated for 3 hours at RT. Wells were washed and probed with asTF-specific detection antibody (RabMab1) conjugated to HRP for 2 hours at RT. TMB substrate was then added and plate was incubated in the dark for 60 minutes; reaction was stopped with sulfuric acid and the plate was read at 450 nm. Serial dilutions of recombinant asTF in mouse plasma were used to generate a concentration curve in each run.

### Tissue harvesting and histological analyses

Harvested tumor specimens were fixed in formalin overnight prior to paraffin embedding. Paraffin-embedded tumors were sectioned (4 μm), baked for 1 hour at 63°C, and rehydrated. Sections were placed in Antigen Retrieval Citra Solution (BioGenex) for 20 minutes in a pre-heated steamer at 95°C; afterwards, slides were allowed to cool and rinsed in wash buffer (Dako). Slides were blocked for 12 min with a blocking cocktail, washed, and incubated with the primary antibodies: rabbit polyclonal anti-CD31 (Abcam) and anti-CD206 (Abcam); anti-β1 (R & D Systems), and anti-α6 (R & D Systems) were applied for 3 hours, slides washed and incubated with appropriate secondary antibodies for 30 minutes and Vectashield/DAPI. Ki67 staining was carried out as previously described [8]; staining for E-cadherin and N-cadherin was performed using rabbit monoclonal antibodies 24E10 and D4R1H, respectively (both from Cell Signaling); staining for fibrin(ogen) was carried out using rabbit polyclonal antibody A0080 (Dako) that was previously characterized and shown to recognize human and murine fibrin(ogen) [44]. To detect apoptosis, TUNEL was performed using ApopTag Peroxidase *In Situ* Apoptosis Detection kit (EMD Millipore Cat. No. S7100). Deparaffinized tissue sections were treated with proteinase K (Thermo Scientific Cat. No. 17916) 200 μg/mL for 15 min at RT, endogenous peroxidase quenched with 3.0% H_2_O_2_, stained as per the manufacturer's protocol and developed with DAB (DAKO). Images were captured using BZ-9000 BIOREVO (Keyence) and analyzed using BZ-II analyzer and Image J.

### Statistics

Mean values were compared using Student's *t*-test between two groups, and for multiple group comparisons one-way ANOVA was used; *p* < 0.05 was considered significant. For statistical analyses, equal variance test was run, and in the cases where it failed, One Way ANOVA on Ranks Test was run along with Dunn's test for all pairwise comparisons. All statistical analyses were performed using SigmaPlot v12.5. Data are presented as the mean ± standard deviation.

## SUPPLEMENTARY MATERIALS FIGURES


