# Supplementary material for: Antibody-based targeting of alternatively spliced tissue factor: a new approach to impede the primary growth and spread of pancreatic ductal adenocarcinoma
Source: Oncotarget. 2016 Mar 7;7(18):25264–75. doi: 10.18632/oncotarget.7955 (PMC5041902; doi:10.18632/oncotarget.7955)
Supplement: Supplementary file 1 [file oncotarget-07-25264-s001.pdf]

Antibody-based targeting of alternatively spliced tissue factor: a new approach to impede the primary growth and spread of pancreatic ductal adenocarcinoma

Supplementary Materials

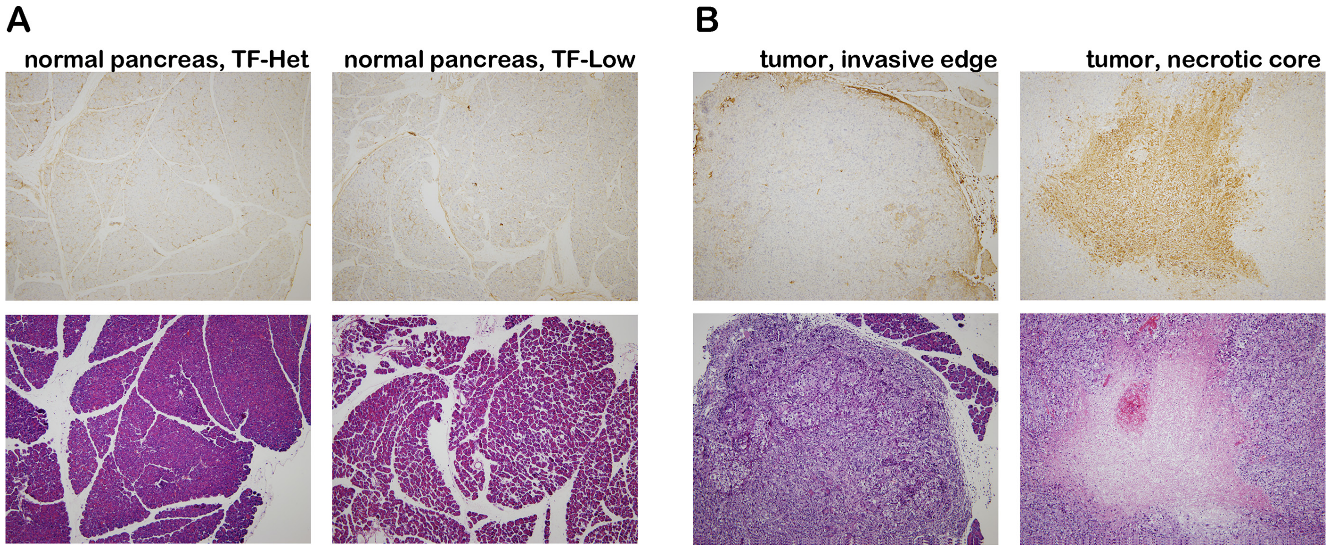

Supplementary Figure 1: Fibrin(ogen) staining, normal pancreas (A) and tumor tissue (B). Top panels, fibrin(ogen) wherein brown indicates positive immunostaining; lower panels, H&E. Original magnification: 10X.
